# A regulatory hydrogenase gene cluster observed in the thioautotrophic symbiont of *Bathymodiolus* mussel in the East Pacific Rise

**DOI:** 10.1038/s41598-022-26669-y

**Published:** 2022-12-23

**Authors:** Ajit Kumar Patra, Maëva Perez, Sook-Jin Jang, Yong-Jin Won

**Affiliations:** 1 Division of Ecoscience, Ewha Womans University, Seoul, Republic of Korea; 2 Department of Biological Sciences, Université de Montréal, Montreal, Canada; 3 Ocean Science and Technology Institute, Inha University, Incheon, Republic of Korea

**Keywords:** Evolutionary ecology, Genomics, Comparative genomics, Genome evolution

## Abstract

The mytilid mussel *Bathymodiolus thermophilus* lives in the deep-sea hydrothermal vent regions due to its relationship with chemosynthetic symbiotic bacteria. It is well established that symbionts reside in the gill bacteriocytes of the mussel and can utilize hydrogen sulfide, methane, and hydrogen from the surrounding environment. However, it is observed that some mussel symbionts either possess or lack genes for hydrogen metabolism within the single-ribotype population and host mussel species level. Here, we found a hydrogenase cluster consisting of additional H_2_-sensing hydrogenase subunits in a complete genome of *B. thermophilus* symbiont sampled from an individual mussel from the East Pacific Rise (EPR9N). Also, we found methylated regions sparsely distributed throughout the EPR9N genome, mainly in the transposase regions and densely present in the rRNA gene regions. CRISPR diversity analysis confirmed that this genome originated from a single symbiont strain. Furthermore, from the comparative analysis, we observed variation in genome size, gene content, and genome re-arrangements across individual hosts suggesting multiple symbiont strains can associate with *B. thermophilus*. The ability to acquire locally adaptive various symbiotic strains may serve as an effective mechanism for successfully colonizing different chemosynthetic environments across the global oceans by host mussels.

## Introduction

In chemosynthetic habitats such as hydrothermal vents and cold seeps, deep-sea invertebrates rely upon the mutualistic symbiotic partnership with chemoautotrophic bacteria^1^. *Bathymodiolus* mussels are among the most prevalent deep-sea animals that contribute to the deep-sea chemosynthetic habitats^1,2^. *Bathymodiolus* mussels feed on the nutrients provided by the symbiotic bacteria residing inside specialized cells of the gill tissues^3,4^. These endosymbiotic bacteria generate organic carbon by fixing CO_2_ dissolved in the seawater. This reaction is powered by the chemical energy generated from the oxidation of reduced compounds (methane, hydrogen sulfide and so on) which are also sourced from the environment^5–7^.

Bathymodiolonae mussels have broad geographic distributions and occupy a wide range of chemosynthetic habitats along global mid-ocean ridge system, in back arc basins, in hydrocarbon seeps along continental margins and organic falls such as whale carcasses and wood-falls^8–14^. Like other deep-sea invertebrates such as siboglinid tubeworms^15^ and lucinid clams^16^, *Bathymodiolus* mussels acquire their chemosynthetic symbionts horizontally from the vicinity^15,17^ during their early juvenile stage^18,19^. Indeed, while studies of several *Bathymodiolus* species such as *B. childressi*, *B. azoricus*, *B. heckaerae*, and *B. puteoserpentis* have documented the occurrence of gill symbionts during the early life stages of the mussels^20,21^ these symbionts were not observed in either the gonads or gametes of males and females^19,22,23^. A large body of work has demonstrated that most *Bathymodiolus* mussels can host methane-oxidizing and sulfur-oxidizing (SOX) bacteria in their gills, and that multiple SOX symbiont strains can co-exist within a single host^24^. It was hypothesized that the capacity of host mussels to acquire and exploit locally adapted species and strains of symbiotic bacteria was the reason for such successful distribution of host mussels in diverse deep-sea chemosynthetic habitats globally^15,17^. However, it is still impossible to directly culture the symbionts and experiment with them. In this context, comparative genomic analyses of *Bathymodiolus* symbionts can be an alternative to understand diverse symbionts’ metabolic ability with respect to the different environment and locations.

The SOX symbionts in mussels are closely related to the free-living Gammaproteobacteria SUP05^25^. To date the SOX symbiont genomes of *B. azoricus*^26^, *B. septemdierum*^8^, *B. heckerae*^10^, *B. puteoserpentis*^10^, *B. brooksi*^10^, *B. thermophilus*^12^, and an undescribed *Bathymodiolus* species from the Mid-Atlantic Ridge (*B*. sp. 5 South)^10^ have been reported (Table 1). Genomic analyses suggest that these symbionts use carbon fixation, sulfur oxidation, nitrogen, and hydrogen metabolism to harness energy. However, to adapt in variable geochemical conditions of the surrounding environments, some metabolic genes or pathways (for hydrogen and nitrogen metabolism) are either present or absent in symbionts of the same mussel species, as previously studied in *B. septemdierum* (from Myojin knoll, Japan)^8^ and *B. azoricus* (from Lucky Strike vent field)^27^. Hydrogen as an potential inorganic energy source other than sulfur or methane was first reported in symbionts of *B. puteoserpentis* from Logatchev hydrothermal vent field^28^. In the same study Petersen et al., 2011 found the hydrogenase gene in *B. thermophilus* from the Axial Dome vent of Pacific-Antarctic Ridge region^28^. However, previously studied symbiont genomes of *B. thermophilus* from the EPR9N regions do not possess any hydrogenase genes^10,12^. Although, it has been studied that hydrogen abundancy in EPR9N regions is similar to the Logatchev hydrothermal vent regions^29^. In this study, we found that symbiont genome of *B. thermophilus* studied from the EPR9N region encodes a hydrogenase cluster with additional hydrogenase subunits.

**Table 1:** General information about *Bathymodiolus* thiotrophic symbiont genomes used for phylogenomic analyses.

*Bathymodiolus* species	Name of assembly	Location	Latitude	Longitude	References
*B. azoricus*	SOX Menez Gwen	Atlantic Ocean	37°50′40.0"N	31°31′08.0"W	^10^
*B. azoricus*	BazSymA	Atlantic Ocean	37°45′34.7"N	31°38′15.7"W	^26^
*B. azoricus*	BazSymB	Atlantic Ocean	37°50′40.8"N	31°31′10.2"W	^26^
*B. azoricus*	SOX ET2 1586I	Atlantic Ocean	37°17′21.2"N	32°16′32.2"W	^10^
*B. brooksi*	BBROOK1789B*	Chapopote, GoM	21°54′00.2"N	93°26′07.4"W	^10^
*B. brooksi*	BROOK1789C*	Chapopote, GoM	21°54′00.2"N	93°26′07.4"W	^10^
*B. brooksi*	BBROOKSOX*	Chapopote, GoM	21°54′00.2"N	93°26′07.4"W	^10^
*B. heckerae*	BHECKSOX*	Chapopote, GoM	21°54′00.2"N	93°26′07.4"W	^10^
*B. heckerae*	BHECKSOX2*	Chapopote, GoM	21°53′58.8"N	93°26′07.2"W	^10^
*B. puteoserpentis*	BPUTEOSOX	Logatchev, MAR	14°45′18.0"N	44°59′16.0"W	^10^
*B. septemdierum*	Myojin Knoll	Myojin knoll, Japan:IBA	32°06′14.8"N	139°13′09.5"E	^8^
*B.* sp. 5 South	BCLUESOX	5° South, Clueless, MAR	4°48′11.8"S	12°22′18.5"W	^10^
*B.* sp. 5 South	BTURTLESOX	5° South, Wide Awake, MAR	4°48′33.5"S	12°22′27.8"W	^10^
*B. thermophilus*	THERMOS	EPR	9°50′22.6"N	104°17′32.0"W	^10^
*B. thermophilus*	THERMOT	EPR	9°50′22.6"N	104°17′32.0"W	^12^
*B. thermophilus*	BAT/CrabSpa'14	EPR	9°30′00.0"N	104°10′12.0"W	^10^
*B. thermophilus*	BTHERMOSOX	EPR	9°50′22.6"N	104°17′32.0"W	^10^
*B. thermophilus*	EPR9N	EPR	9°49′12.0"N	104°18′00.0"W	This study

*GoM* Gulf of Mexico, *EPR* East Pacific Rise, *MAR* Mid Atlantic Ridge, *IBA* Izu-Bonin Arc.

*From the cold seep habitat. Remaining assemblies are from the hydrothermal vent habitat.

The SOX symbionts in mussels from different geographical locations may possess distinct genome structures, accessory genes of ecological significance, and foreign genetic material indicative of their exposure to habitat-specific viromes^30^. Also, symbiont genome possess methylated DNA that may methylate host genome recognition sites^31^ and can respond to the environmental stresses^32^. However, the fragmented, incomplete, and contamination-prone nature of most of the published genome draft assemblies complicates comparative investigations of *Bathymodiolus* SOX symbiont accessory genome, structural rearrangements (such as gene duplications, translocations, or inversions) and hinders the study of mobile genetic elements and horizontal gene transfer. Therefore, in this study, we present a high-quality complete genome of the SOX symbiont associated with a specimen of *B. thermophilus* from the 9 N hydrothermal vent fields of East Pacific Rise (hereafter the symbiont genome referred to as EPR9N). We sequenced this symbiont genome using PacBio sequencing technology and assembled it with a hierarchical genome-assembly process (HGAP3) pipeline.

## Materials and methods

### Sample collection and DNA extraction

One *Bathymodiolus thermophilus* mussel specimen was collected from the Choo Choo site of the East Pacific Rise in 2000 (Dive number: Alvin 3540; latitude 9.82 N and longitude 104.30 W). After reaching onboard, the whole body was immediately preserved at − 80 °C, transported to the laboratory in dry ice, and stored at − 80 °C until further processing. To get high molecular weight DNA, the genomic DNA was extracted and purified from a small piece of the mussel gill tissue using the CHAOS buffer protocol and the phenol–chloroform method^33^.

### Symbiont genome sequencing and assembly

Sequencing was performed using PacBio RS II SMRT sequencing technology (Pacific Biosciences, Menlo Park, CA, USA). An average of 20 kb insert SMRTbell library was constructed and sequenced, yielding 122,925 reads (126 X average genome coverage). De novo assembly was conducted using the hierarchical genome-assembly process (HGAP3) pipeline of the SMRT Analysis v2.3.0^34^ using PacBio CLRs. After gap closing, the newly generated contig served as a reference to which raw PacBio reads were mapped using the resequencing module of the SMRT protocol^34^. Finally, a consensus assembly was finalized with Trycycler v0.5.3^35^ from the multiple input assemblies generated by HGAP3 pipeline^34^, Flye v2.9^36^ and Unicycler v3.0^37^ assemblers. Plasmid sequences are screened from the assemblies generated by PlasFlow^38^ (Table S11).Assembly quality and completeness were assessed with Quast v5.0.0^39^ and CheckM v1.0.18^40^ based on 280 Gammaproteobacteria specific single copy marker genes.

### Assessment of symbiont diversity in the DNA library

The sequencing and assembly of multiple strains of the SOX symbiont would result in a chimeric genome which may not be representative of a true symbiont chromosome. To verify that EPR9N was assembled from a monoclonal symbiont library, we assessed the genome wide SNP frequency distribution and the genetic diversity of the clustered regularly interspaced short palindromic repeats (CRISPR) marker. One CRISPR array was identified in the EPR9N symbiont genome and its spacer sequences extracted with CRISPR-Cas +  + webtool^41^. Following the protocol in^42^, we characterized the CRISPR haplotypes within our symbiont sequencing library by near matching (identity threshold = 80%) the spacers sequences to the CRISPR-containing PacBio reads using a modified version of the script call_CRISPR_haplotypes.py (available at https://github.com/maepz/CRISPR_distance/tree/master/manuscript_data_and_downstream_analyses_scripts/haplotype_detection_pipelines).

### Gene prediction and annotation

We identified protein-coding genes using Prodigal v2.6.1^43^. The predicted CDSs were BLAST-searched against UniProt, Pfam, and COG databases to gain insights into molecular functions and family classification of predicted genes. Signal peptides and transmembrane helices were predicted using SignalP v4.1^44^ and TMHMM v2.0^45^. rRNA, tRNA, and other miscellaneous features were predicted using RNAmmer v1.2^46^, tRNAscan-SE v1.21^47^, and Rfam^48^, respectively. KEGG orthology (KO) numbers and functions were retrieved using BlastKOALA online^49^.

### Methylation and mobilome analysis

The DNA base modification analysis was detected with SMRTAnalysis_2.3.0.140936. The mobile genetic elements were identified by webtools of ISfinder (https://isfinder.biotoul.fr/)^50^, IslandViewer4 (https://pathogenomics.sfu.ca/islandviewer))^51^, and PHASTER (https://phaster.ca/)^52^.

### Phylogenomic and comparative genome analyses

For phylogenomic analyses, two vesicomyid symbiont genomes of *Candidatus* Ruthia endofausta and *Candidatus* Ruthia magnifica^53^ were used as outgroups. We extracted single-copy orthologous genes (SOGs) from the outgroup and all available *Bathymodiolus* sp. SOX symbiont genomes (Table 1) with Orthofinder v2.5.4^54^, cleaned the sequences with trimAl v1.4.1^55^, and built a maximum likelihood (ML) tree using the ‘WAG’ general matrix model in IQ-TREE v2.2.0_beta^56^. The phylogenies of hydrogenase genes (HupUVLS) were analyzed using ML method with the JTT matrix-based model in MEGA-X^57^. The online iTOL tool^58^ was used to visualize the resulting ML tree. We used Anvi’o’s Pangenomics, Phylogenomics, and average nucleotide identity (ANI) of *Spiroplasma* genomes workflow for comparative genome analysis among *B. thermophilus* symbiont genomes^59^. We used Mauve v20150226^60^ and GRIMM web server^61^ to detect structural variation across *B. thermophilus* symbiont genomes. The fragmented *B. thermophilus* symbiont genome assemblies were beforehand aligned to EPR9N and their contigs sorted with D-GENIES webtool^62^. The 49 CRISPR spacers identified in EPR9N were searched in other *B. thermophilus* symbiont genome assemblies with blastn by blast + v2.6.0^63^ (e-value cutoff = 1E10^−6^). Comparisons of hydrogenase gene clusters were visualized by clinker and clustermap.js v1.32^64^. Hydrogenase operon was detected by Operon-mapper web server^65^. Hydrogenases were classified using HydDB web server^66^.

## Results

### EPR9N symbiont genome assembly and annotations

**Figure 1:**
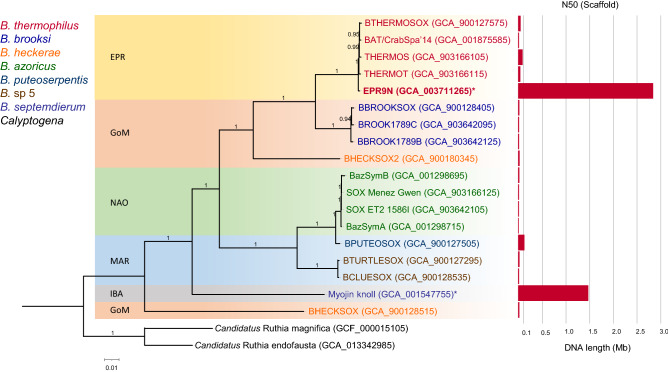
Maximum likelihood phylogeny of single-copy-orthologous genes for deep-sea mussels’ thiotrophic gill endosymbionts. Deep-sea clam symbiont genomes of *Candidatus* Ruthia magnifica and *Candidatus* Ruthia endofausta were used as outgroup. Legends represent the host *Bathymodiolus* species and outgroup *Calyptogena*. Location of mussel symbionts are presented in background-colored boxes. *EPR* East Pacific Rise, *GoM* Gulf of Mexico, *NAO* North Atlantic Ocean, *MAR* Mid Atlantic Ridge, *IBA* Izu-Bonin Arc, Japan. Assembly ID of each symbiont genome is presented in parentheses. The bar chart shows each symbiont genome assembly’s N50 (Scaffold) values. *Represent the complete and circular genomes.

From one single-molecule real-time (SMRT) cell, we obtained 122,925 subreads with an N50 value of 12,539 bp. The longest contig generated by resequencing module of the SMRT protocol was confirmed as circular by Trycycler^35^. The final EPR9N assembly was a 2,832,685 bp circular contig with a GC content of 38.5%. Its mean coverage was 126 X. Assessment of genome completeness based on 280 proteobacteria-specific single copy marker genes resulted in a completeness score of 97.19% with a low level of contamination (1.99%) (Table 2). EPR9N is the most complete *B. thermophilus* gill symbiont genome to date (Table 2) and one of two chromosome level assemblies for *Bathymodiolus* SOX symbionts: the other belongings to *B. septemdierum* symbionts (Fig. 1). Except for the assembly BPUTEOSOX (*B. puteoserpentis* symbionts), the other *Bathymodiolus* mussel symbionts genomes have low contiguity (scaffold N50 < 0.1 Mb) (Fig. 1). Amongst *B. thermophilous* symbionts, assembly sizes varied from 2.25 Mb (BTHERMOSOX) to 3.09 Mb (BAT/CrabSpa'14). Among all predicted genes, 1,968 genes were functionally annotated by Pfam database, while the remaining 99 genes were unclassified or classified as from the unknown protein domains (Table S4). Predicted proteins were assigned to Clusters of Orthologous Groups numbers and Protein Families domains by querying their sequences against the COG database through the Anvi’o pipeline^59^.

**Table 2:** Genome completeness, contamination, and assembly statistics of *B. thermophilus* symbionts.

Name of assembly	Completeness	Contamination	GC	Genome size (bp)	Ambiguous bases	Scaffolds	Longest scaffold	N50 (scaffolds)
EPR9N	97.19	1.99	38.57	2,832,685	0	1	2,832,685	2,832,685
BTHERMOSOX	97.85	0.66	38.40	2,249,231	0	161	144,199	43,780
THERMOS	97.19	0.66	38.19	2,414,318	95	100	347,385	85,091
THERMOT	97.85	0.99	38.42	2,333,609	100	154	126,494	39,115
CrabSpa'14	96.96	6.75	37.74	3,088,407	14,424	1281	61,812	10,694

### Symbiont genetic homogeneity in EPR9N library

One large CRISPR region (length = 3544 bp) with 49 spacers (average length = 34.6 bp) was identified in EPR9N (Table S1). Of the 263 subreads which mapped to the CRISPR locus, 40 contained the whole CRISPR array (including the leader sequence and 5’ neighboring Fic gene). These 40 sequences were identical in their spacer content and no arrays with putative new spacers or missing spacers were observed amongst all the mapped subreads. This result indicates that the libraries used to assemble EPR9N were monoclonal and thus EPR9N circular contig is biologically sound and representative of the chromosome of a SOX symbiont.

### Genome similarity and structural variation

The average nucleotide identity (ANI) of EPR9N was 98% to other *B. thermophilus* symbiont genomes and 77–84% to other *Bathymodiolus* SOX symbiont genomes (Table S3). While the genome-wide ANI was high amongst *B. thermophilus* symbionts, large ANI variations were observed across genes. Indeed, the genome fraction with high identity to EPR9N (nucleotide identity > 75%) was at most 76% with BTHERMOSOX and at least 64% with BAT/CrabSpa'14 (Fig S1) while sequences without any match to EPR9N genome represented 14 to 23% of the other *B. thermophilus* associated symbiont genomes (Fig. S1).

Furthermore, genome alignment and gene rearrangement analyses showed that the genomes of *B. thermophilus* symbionts are structurally distinct and are distinguished by translocations and/or inversions events. Indeed, with reference to EPR9N, all but BAT/CrabSpa'14 *B. thermophilus* symbionts genomes show evidence of reversal events (Fig. S2).

### Phylogenomic and comparative genomics

**Figure 2:**
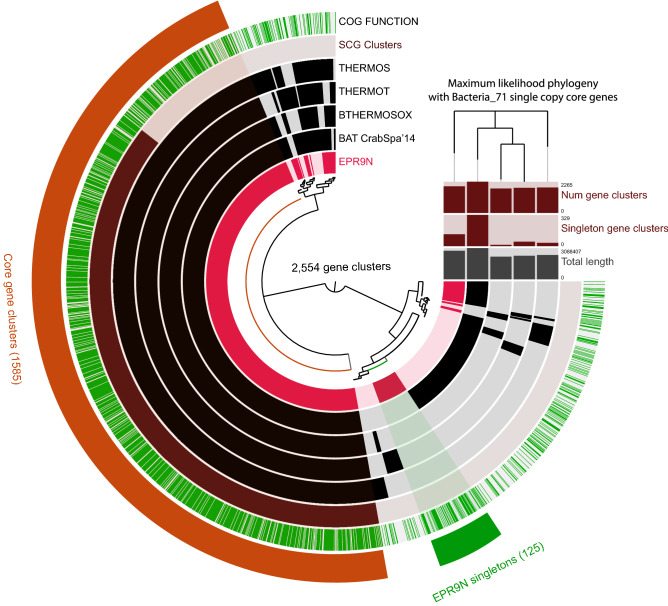
Pangenomic comparison of *B. thermophilus* thiotrophic symbiont genomes. The inner dendrogram shows hierarchical relationships among these clusters based on their distribution across genomes. Within layers, dark colors indicate the presence of a gene cluster, while light colors indicate absence. Different gene cluster groups and their abundances are highlighted outside the pangenome graph, including core gene clusters among all *B. thermophilus* symbiont genomes (red) and EPR9N symbiont strain specific gene clusters (green).

Phylogenetic analyses with 541 single-copy orthologous genes (SOGs) indicated that the *B. thermophilus* thiotrophic symbionts form a single cluster and are closely related to the cluster of *B. brooksi* thiotrophic symbionts from Gulf of Mexico (Fig. 1). All *B. thermophilus* symbiont genomes shared 2554 core gene clusters consisting of 9,365 genes (Fig. 2, Table S5). EPR9N, BAT/CrabSpa'14 and THERMOT contained 125, 131 and 34 unique genes, respectively (Fig. 2, Table S5). The majority of these unique genes did not have clusters of orthologous genes (COG) annotations. The remaining annotated genes include various putative phage and other mobile element remnants (i.e., repeats-containing genes, reverse transcriptases, and transposases), genes for defense mechanism (restriction endonucleases, type I restriction-modification system genes, DNA methylase subunits, RTX toxin-related, and a CRISPR/Cas system (in BAT/CrabSpa'14)). Unique genes in EPR9N also included genes for energy production and conversion such as Ni/Fe-hydrogenases, hydrogenase maturation factors, chaperons like heat shock protein HSP90, rubredoxins (a stress regulating protein) and uncharacterized proteins containing known functional domains (Table S5).

### Energy metabolism

**Figure 3:**
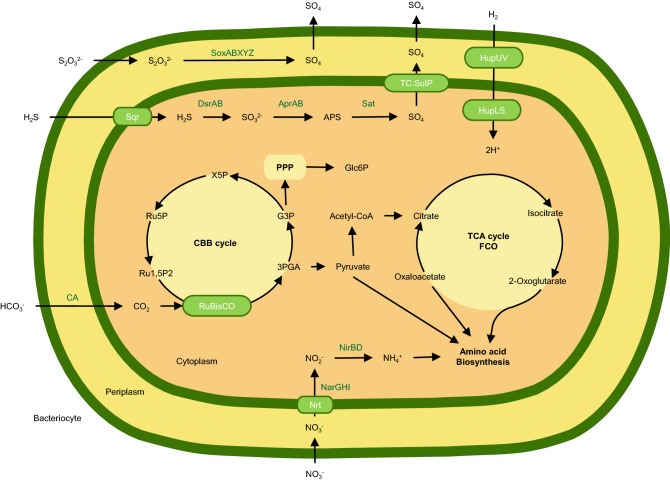
Schematic diagram of key metabolism found in *B. thermophilus* EPR9N symbiont genome. *FCO* First carbon oxidation, *Glc6P* α-D-Glucose-6-phosphate, *PPP* Pentose Phosphate Pathway, *G3P* Glyceraldehyde-3-phosphate, *3PGA* 3-Phospho-glyceric acid, Ru1, *5P2* Ribulose 1,5-bisphosphate, *Ru5P* Ribulose 5-phosphate, *X5P* Xylulose 5-phosphate, *Nar* Nitrate reductase, *Nir* nitrite reductase, *Hup* Ni/Fe hydrogenase. Subcellular localization of bacterial proteins for constructing schematic metabolic pathway were predicted by PSORTb 3.0 webtool^93^.

EPR9N encodes genes for the autotrophic CO_2_-fixation by Calvin-Benson-Bassham (CBB) cycle and the TCA cycle. While key genes for the CBB cycle are found, genes like sedoheptulose-7-phosphatase and fructose-1,6-bisphosphatase are not observed as previously reported^12^. But an alternative gene to complement the CBB pathway (6-phosphofructokinase) was found in the genome. TCA cycle has genes for first carbon oxidation (FCO), i.e., from oxaloacetate to 2-oxoglutarate (Fig. 3). Several other previously reported genes including malate dehydrogenase and succinate dehydrogenase^11^ were not found in EPR9N. This genome as well as other *B. thermophilus* symbiont genomes encodes two complete sets of genes for the SOX system (soxABXYZ), dissimilatory sulfate reduction and oxidation (sat, aprAB, dsrAB genes), assimilatory nitrate reduction, and dissimilatory nitrate reduction (Fig. 3, Tables S4 and S5)^12^.

### Hydrogen metabolism

**Figure 4:**
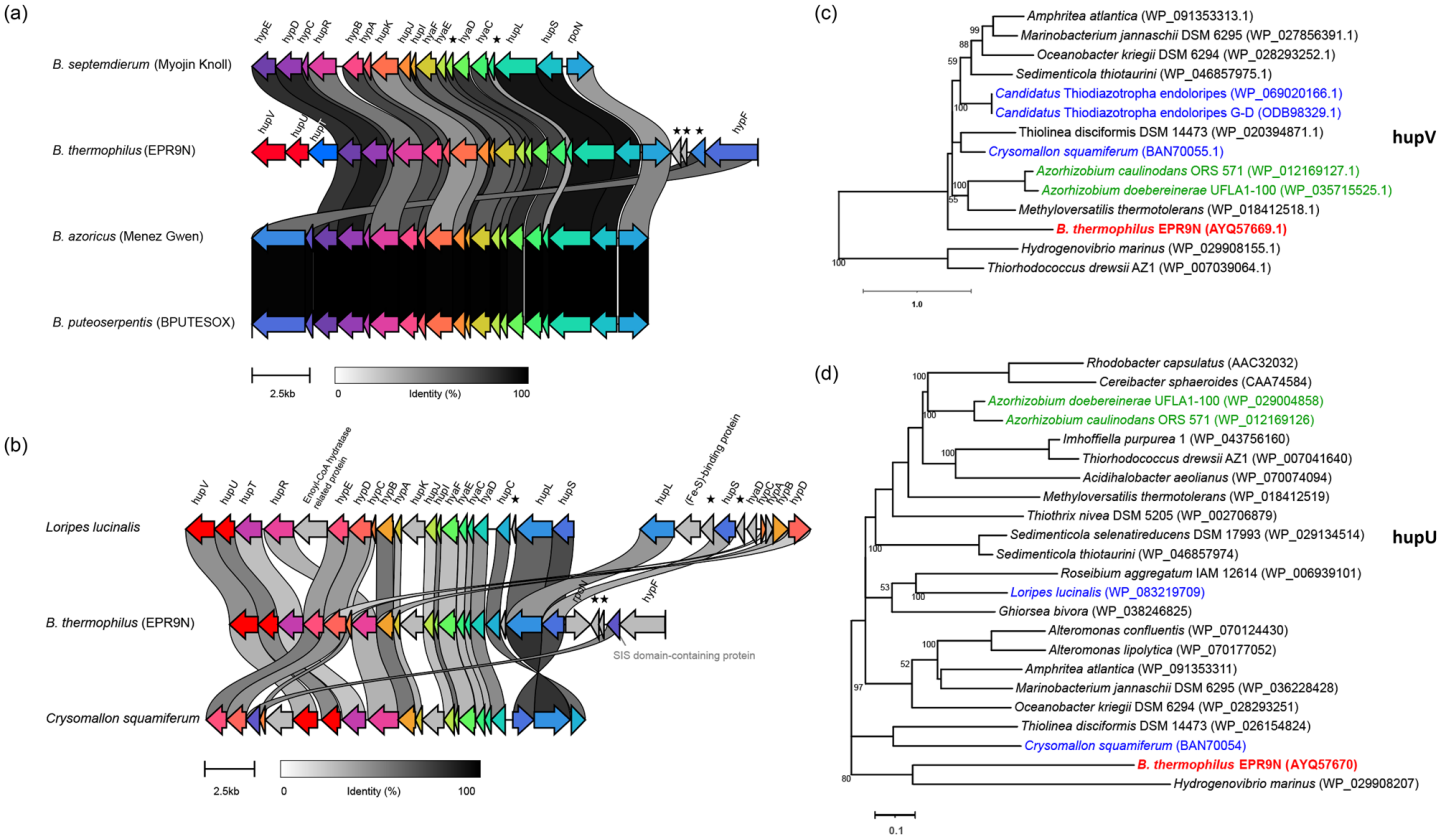
Schematic visualization of hydrogenase cluster regions in (**a**) *Bathymodiolus* mussel thiotrophic symbiont genomes, (**b**) symbiont genomes of *Loripes lucinalis, B. thermophilus* EPR9N, and *Crysomallon squamiferum*. *Loripes lucinalis* symbiont genome possesses two distinct hydrogen clusters. Star marks are hypothetical genes. Phylogenies of H_2_-signaling hydrogenases (**c**) HupV and (**d**) HupU genes, respectively. The phylogenetic trees were inferred by using the maximum likelihood method with bootstrap values on the tree branches (only over 50 are shown). The taxa in green are of the symbionts of legume host *Sesbania*, in blue are the symbionts of marine gastropods, and in bold red are the symbiont of *B. thermophilus* EPR9N. GenBank accession numbers of proteins are presented in parentheses.

Interestingly, a regulatory hydrogenase cluster, identified as an operon (Table S8) was exclusively found in EPR9N, but not in the other *B. thermophilus* symbiont strains. (Table S5). However, the hydrogenase cluster was present in a few other *Bathymodiolus* symbionts with some variation in gene order and content. The arrangement pattern for the hypABCDE, hupIJKLRS, hyaCDEF and rpoN genes was conserved across the symbiont genomes, whereas hypF and another hypothetical gene appeared translocated in the EPR9N genome in comparison to *B. azoricus* (Menez Gwen) and *B. puteoserpentis* (BPUTESOX). BLASTP homology inferences showed that EPR9N hupLS and hypDE genes are > 80% identical to these of other *Bathymodiolus* mussel symbiont strains (Fig. 4a). In contrast, the same homologous genes showed > 97% identity between the *B. azoricus* (Menez Gwen) and *B. puteoserpentis* (BPUTESOX) indicating that both symbionts are closely related, which is also evident from the phylogenomic analysis. The phylogenies of hupLS genes reveal the pervasive distribution of these genes across freely living bacteria, legume symbionts and the symbionts of deep-sea invertebrates (i.e., scaly-foot snails, clams, bivalves, and tubeworms) (Fig. S3a,b). Furthermore, two additional subunits of H_2_-sensing hydrogenase gene (hupUV) along with a histidine kinase (hupT) gene were exclusively found in the EPR9N genome among the *Bathymodiolus* mussel symbionts (Fig. 4a). The hupUV and hupT genes of EPR9N strain are homologous to those of the symbiont of lucinid bivalve *Loripes lucinalis* living in low intertidal to about 20 m depth of European and Mediterranean coasts and the symbiont of scaly-foot snail *Crysomallon squamiferum* living in deep-sea hydrothermal vents of the Indian Ocean with similar gene order (Fig. 4b). BLASTP inference from the hydrogenase cluster gene-rearrangement analysis shows that hupUV genes of EPR9N is > 77% identical to the scaly-foot snail symbiont (Fig. 4b). An extended comparison of the hydrogenase cluster has revealed that the lucinid bivalve has two distinct hydrogenase clusters with a set of hydrogenase subunits (hupLS) in each cluster (Fig. 4b). The hydrogenase cluster in EPR9N genome contains both [NiFe] Group 2b and and 1d hydrogenases (Table S9). Phylogeny analysis confirmed that hupUV genes of EPR9N strain are distantly related to the symbionts of *L. lucinalis* and *C. squamiferum*, and to the free-living bacteria *Hydrogenovibrio marinus* (Fig. 4c,d).

### Interaction with the host

Our study shows that EPR9N contains a variety of toxin and toxin-antitoxin genes, which are also found in other *B. thermophilus* symbiont genomes. Toxin genes such as insecticide toxin hcdB, putative AbiEii toxin, RTX toxin, and antitoxin genes such as Antidote-toxin recognition MazE, Antitoxin Phd_YefM, MraZ protein, and ParE toxin of type II toxin-antitoxin system were found. We found various genes coding for Coagulation factor, Cadherin, Phage integrase, Transglycosylase, Transpeptidase, RHS repeat, etratricopeptide repeat, Type II and IV secretion system proteins, Colicin V, hemolysin-D, which could be important for symbiont to attach and invade and colonize the host tissue (Tables S4 and S5).

### Interaction with viruses

The CRISPR/Cas immunity was present in all *B. thermophilus* thiotrophic symbiont genomes and several assemblies appear to contain multiple CRISPR loci (Table S1). Unfortunately, because of the nature of the CRISPR array (short reads do not assemble well over repeats), CRISPR loci were too fragmented to do in-depth characterization. We nonetheless performed BLASTN searches of EPR9N CRISPR spacers against the other *B. thermophilus* thiotrophic symbiont genome assemblies and found matches for nine of the 49 spacers (Table S2). Interestingly, neighboring spacers different from the ones in the reference array were often found in the CRISPR containing contigs of the *B. thermophilus* symbiont genomes. Spacer 6 even had multiple matches within the BAT/CrabSpa’14 assembly. This could reflect that the CRISPR immunity in this bacterial species is remarkably active, but further investigation of the CRISPR/Cas evolution and the dynamics of spacer acquisition would be needed to shed further light on the symbiont-virus interactions.

### DNA methylation

**Figure 5:**
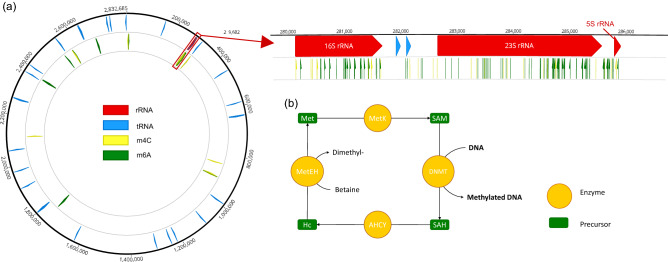
(**a**) The methyl modification regions in *B. thermophilus* EPR9N genome. Zoomed portion shows m4C and m6A base modifications in the rRNA region. (**b**) Enzymatic pathway involved in DNA methylation process. *DNMT* DNA methyltransferase, *AHCY* S-adenosyl-homocysteine hydrolase, *Met* Methionine, *Hc* Homocysteine, *SAH* S-adenosyl-homocysteine, *SAM* S-adenosyl methionine.

We found the methyl modification in the EPR9N genome corresponding to the m4C and m6A DNA methylation (Fig. 5a). Out of 619 m4C and m6A base modifications, 157 high quality DNA base modification signals (QV ≥ 30) are found in dense patches in rRNA, and in transposase regions. All bacterial methyltransferase genes required for DNA methylation were present in the EPR9N genome (Fig. 5b).

### Mobilome

A phage infection region was detected in the EPR9N genome comprising an incomplete ~ 8.5 kb prophage region consisting of 5 phage hit proteins with two bacterial and 2 hypothetical proteins (Fig. S4). Notably, type II toxin-antitoxin system VapC family toxin, tetratricopeptide repeat protein and IS5 family transposase proteins are found in this phage region. Additionally, we have detected genome island regions by IslandViewer4 (Fig. S4 and Table S6). A total of 9 genome island regions were present across the EPR9N genome. Whereas ISfinder detected a total of 58 insertion sequences (IS) of 42 IS classes in the EPR9N genome (Table S7).

## Discussion

While a total of 64 symbiont genomes from ten *Bathymodiolus* mussel species have already been sequenced^10,12,26,67^, the present single contig with high-quality assembly is only the second complete genome of a *Bathymodiolus*-associated SOX symbionts and the first for the species *B. thermophilus*. As such, it represents a valuable resource for further studies on the evolution and biogeography of the deep-sea mussel holobionts.

Symbiont phylogenetic divergence seems correlated to ancient and contemporary geographic locations as the eastern Pacific (the East Pacific Rise and the Gulf of Mexico), Atlantic, and western Pacific symbionts are clustered separately (Fig. 1), as observed in case of phylogenetic relationship of tubeworm symbiont in different locations^68^. For instance, the relatively close genetic distance between the symbionts of *B. brooksi* in the Gulf of Mexico and *B. thermophilus* in the EPR suggests a potential connection between the two locations through ancient pathways in the past. Of course, the possible paths must have disappeared since the geological connection between the north and south Americas. Gene order within the hydrogenase gene cluster further supports this geographical structuring, particularly when the geographic distances between locations are more extended, like the comparison between the Eastern Pacific and the Atlantic symbionts. This pattern is roughly consistent with the phylogeny of *Bathymodiolus* mussel symbionts based on 16S rRNA gene^69^ but the host and symbiont phylogenies are not completely concordant. These phylogenetic discrepancies are likely explained by the horizontal symbiont transmission mode in *Bathymodiolin* mussels^70^. Indeed, like other symbiont-relying deep-sea invertebrates, *Bathymodiolin* mussels acquire their chemosynthetic symbiotic partners from their local surroundings^19,21,71^. Mussel *B. heckrae* acquires multiple thiotrophic symbiont clades^10,72^, one is related to the Gulf of Mexico clade and the other one is distinct from the other mussel symbiont clades (Fig. 1), which shows an exception to the broad geographic relationship between mussels and their thiotrophic symbionts. Nevertheless, low sequence similarity across SOX symbionts associated with different *Bathymodiolus* species occurring from the same locality (*B. brooksi* and *B. heckerae*; Table S3) suggests that the partner choice may be to a certain degree influenced by host specificity and/or local niche partition in the free-living symbiont pool.

*B. thermophilus* symbiont genomes from the EPR showed 98% ANI, suggesting that they belong to the same species according to ANI cutoff values (94–96%)^73–75^. Still, our comparative genomic analyses revealed high variation in genomic structure and gene composition among the different *B. thermophilus* symbionts. Mis-assemblies, incompleteness, and contamination in the fragmented genomes can explain some of the observed differences but we suspect that the most of the accessory pangenome reveals actual SOX strain genetic diversity as observed in other deep-sea mussels^24,27,76,77^, tubeworms^78,79^, clams^80^ and snails^81,82^.

*B. thermophilus* EPR9N symbiont genome possesses two incomplete gene sets for CO_2_-fixation, two complete gene sets for sulfur metabolism, and a gene set for nitrogen metabolism, as previously observed^11,12^. This strain is, however, unique regarding its putative hydrogen oxidation metabolism. Indeed, EPR9N possesses a hydrogenase cluster that is not found in other *B. thermophilus* symbiont strains. The Ni/Fe-hydrogenases encoded in this cluster are required for utilizing hydrogen as an energy source and are found in other invertebrates SOX symbionts^11,28,83^. We found that this cluster is also present in some but not in all *Bathymodiolus* symbionts with variation across symbiont subpopulations for *B. azoricus.* Different Ni/Fe-hydrogenases content was also observed amongst *B. septemdierum* symbionts^8^. Interestingly, in the invertebrate and *Bathymodiolus* symbiont strains which possess it, the hydrogenase cluster is structurally different from that in EPR9N^11,28,83^. Hydrogenase subunits in *Bathymodiolus* symbionts are clustered together with a rubredoxin gene (rpoN), which is required for the biosynthesis of Ni/Fe-hydrogenases^84^ but is absent in the symbiont hydrogenase cluster of the bivalve *Loripes lucinalis* and the scaly-foot snail *Crysomallon squamiferum*. On the other hand, two additional subunits of H_2_-sensing hydrogenase genes, hupUV, along with a histidine kinase gene hupT are only found in EPR9N, *L. lucinalis*^85^, and *C. squamiferum* symbionts^86^. These hydrogenase genes are responsible for regulating H_2_ along with regulatory proteins expressed from the hydrogenase gene cluster^87^. Histidine kinase, together with hupR, forms a two-component regulatory system required for H_2_ induction^87^. The exclusive presence of hupUV genes suggests that the EPR9N symbiont strain has potential to utilize hydrogen sources uniquely among the mussel symbionts. However, further study is needed to assess the presence and activity of hydrogenase clusters in *Bathymodiolus* symbiont populations in deep-sea environments. The same level of sequence divergence across EPR9N, *L. lucinalis* and *C. squamiferum* was observed for the group specific hupUV and hupT genes and the pervasive hupL and hupS genes. This phylogenetic signal suggests hupUV and hupT were not *horizontal*ly acquired in EPR9N exclusively but rather lost in the other sequenced *Bathymodiolus* symbiont strains or missing in their incomplete genome assemblies. The endosymbionts of vesicomyid clams, which are closely related to *Bathymodiolus* symbionts but vertically transmitted, present similar contrasting patterns of gene conservation across species. These differences are tied to key physiological traits (such as anaerobic respiration and dependency on vitamin B12) and are suspected to be adaptations to divergent host-derived or external environmental contexts^88^. Additional sampling of *Bathymodiolus* SOX symbiont strains along with better abiotic characterization of their external and host-associated environment should help us elucidate the evolutive history of the hydrogenase cluster in the mussel symbionts.

DNA methylation in bacteria is involved in diverse defensive biological processes such as DNA mismatch repair, environmental stress responses, or bacteriophages and transposases activity repression^89,90^. Evidence of DNA methylation together with genes required for methylation were found in EPR9N genome but this epigenetic system do not appear to be ubiquitous amongst deep-sea chemosynthetic symbionts. Indeed, the genome of the tubeworm symbiont *Candidatus* Endorifita persephone, which like the *B. thermophilus* symbiont has high host-specificity, does not seem to be methylated and the methyltansferase genes it encodes appear to be inactivated by mobile elements^91^. This raises questions about the role of epigenetics in host-associated and free-living contexts, and about the evolutive consequences of transitioning towards stricter host-specificity on the symbiont ecology.

Finally, we found that many of the genes unique to each *B. thermophilus* symbiont strain putatively originated from phages and mobile elements or were associated with viral defense mechanisms. The CRISPR/cas immunity was present across all *B. thermophilus* symbiont strains and appeared to be functional based on the high diversity and apparent dynamism of spacers. This finding indicates that the symbiont strains in their free-living stage may be under strong selective pressure from the environmental virome and could be more prone to acquire genes through horizontal gene transfer. Presence of genes such as adhesins, cadherins, hemolysin, etc. in EPR9N symbiont genome suggest that the symbiont have capability to attach to host cell, invade and colonize the host tissues as previously studied in the symbiont *B. thermophilus*^92^.

The presence of several ISs and genomic islands suggests that *B. thermophilus* EPR9N symbiont has acquired foreign DNA by horizontal transfer process from the different bacterial populations.

## Conclusions

Here we report an improved complete genome assembly of the deep-sea hydrothermal vent mussel *B. thermophilus* thiotrophic gill symbiont (EPR9N) from the East Pacific Rise, using PacBio sequencing technology with a hierarchical genome-assembly process (HGAP3) pipeline. This symbiont genome is composed of a single circular chromosome which was assembled from a monoclonal symbiont population residing in the gill tissue of a host mussel. The discovery of a hydrogenase cluster with additional hydrogenase subunits in the EPR9N mussel symbiont genome showed that the symbiotic strain may have potential to harness energy efficiently by utilizing H_2_ resource from the vent fluids. Further study with extensive samples from diverse chemosynthetic environments required to understand the evolutionary history of the hydrogenase cluster in EPR9N symbiont genome. The symbiosis study of the host invertebrates and its symbiotic bacteria is essential to understand the chemosynthetic ecosystem. Even so, genomic studies of symbiotic bacteria at both species and population levels are limited. Therefore, the present mussel symbiont genome will serve as a footstep for further comparative genomic analyses investigating adaptive symbiont genome evolution in deep-sea chemosynthetic environments, particularly with regard to environmental niche partitioning, interactions with viruses, and horizontal transfer of accessory genes.

## Supplementary Information

Supplementary Information.

## Data Availability

The genome data was deposited to BioProject with an accession number PRJNA416436 in the NCBI BioProject database (https://www.ncbi.nlm.nih.gov/bioproject/). The BioSample accession number for the symbiont genome is SAMN07956337 and assembly accession number GCA_003711265.1.
