# Supplementary material for: A regulatory hydrogenase gene cluster observed in the thioautotrophic symbiont of Bathymodiolus mussel in the East Pacific Rise
Source: Sci Rep. 2022 Dec 23;12:22232. doi: 10.1038/s41598-022-26669-y (PMC9789115; doi:10.1038/s41598-022-26669-y)
Supplement: Supplementary file 1 — Supplementary Information. [file 41598_2022_26669_MOESM1_ESM.pdf]

Supplementary Materials

A regulatory hydrogenase gene cluster observed in the thioautotrophic symbiont of *Bathymodiolus* mussel in the East Pacific Rise

Ajit Kumar Patra[1], Maëva Perez[2], Sook-Jin Jang[3] and Yong-Jin Won[1*]

[1]Division of Ecoscience, Ewha Womans University, Seoul, Republic of Korea

[2]Department of Biological Sciences, Université de Montréal, Montreal, Canada

[3]Ocean Science and Technology Institute, Inha University, Republic of Korea

*Correspondence: won@ewha.ac.kr

Symbiont genome assembly and plasmid prediction.

Filtered PacBio subreads were used for assembly by all three assemblers. De novo assembly was conducted using the hierarchical genome-assembly process (HGAP3) pipeline of the SMRT Analysis v2.3.032. After gap closing, the newly generated contig served as a reference to which raw PacBio reads were mapped using the resequencing module of the SMRT protocol. Assembly by Flye v2.9 (--pacbio-raw) and Unicycler v3.0 (-l) was used with command for PacBio reads as mentioned by the developers. Then, we used these assemblies to cluster contigs to groups similar contigs together and then reconciled manually validated contig clusters by Trycycler. Assembly statistics generated by three assemblers are presented in Table S10. Plasmids from the assemblies are predicted by PlasFlow are presented in Table S11.

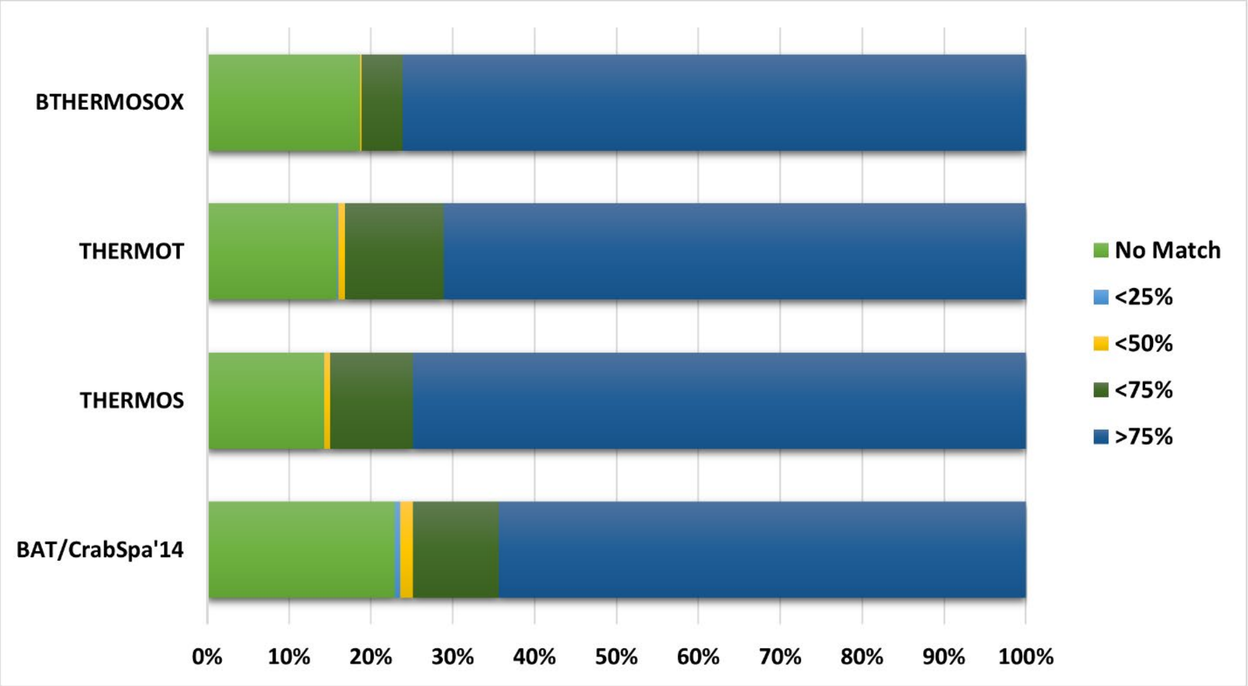

Figure S1 Sequence alignment similarity of *B. thermophilus* thiotrophic symbiont genomes to the EPR9N genome.

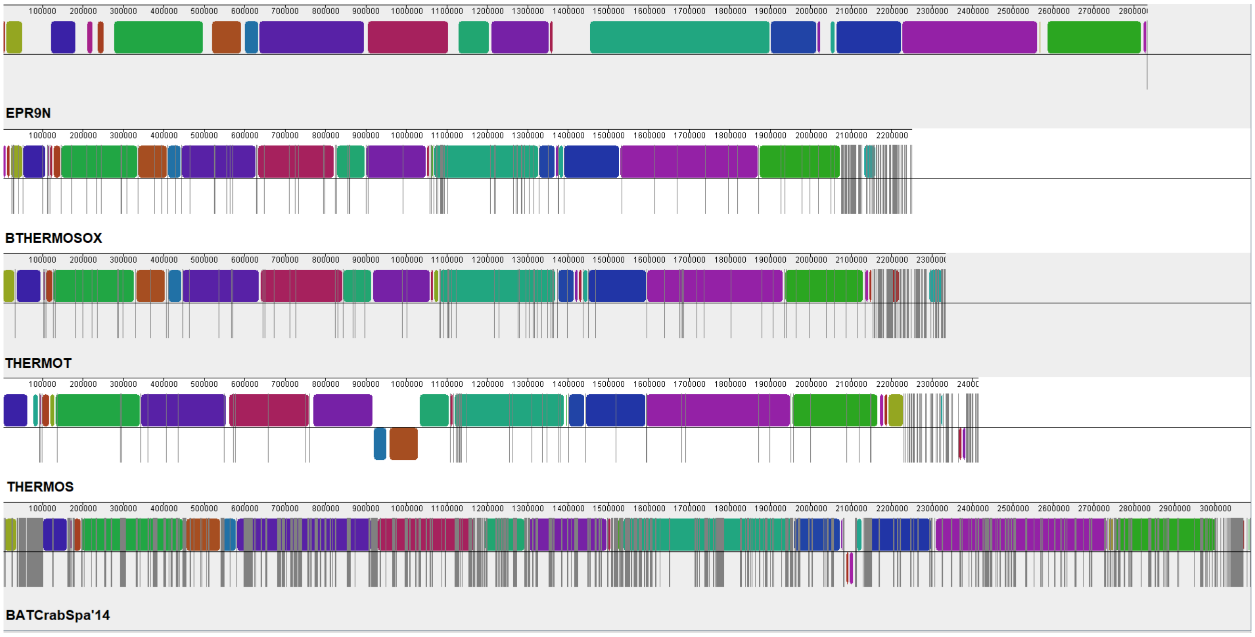

Figure S2. Structural rearrangements between *B. thermophilus* thiotrophic symbiont genome assemblies. Contigs of the fragmented assemblies were sorted and reoriented to match as best as possible the reference EPR9N assembly. Colored fragments indicate locally collinear blocks (LCBs) where sequences are homologous and show no putative structural variation. Fragments under the central horizontal line indicate inversions compared to EPR9N. Contig boundaries of the assemblies are marked with grey bars.

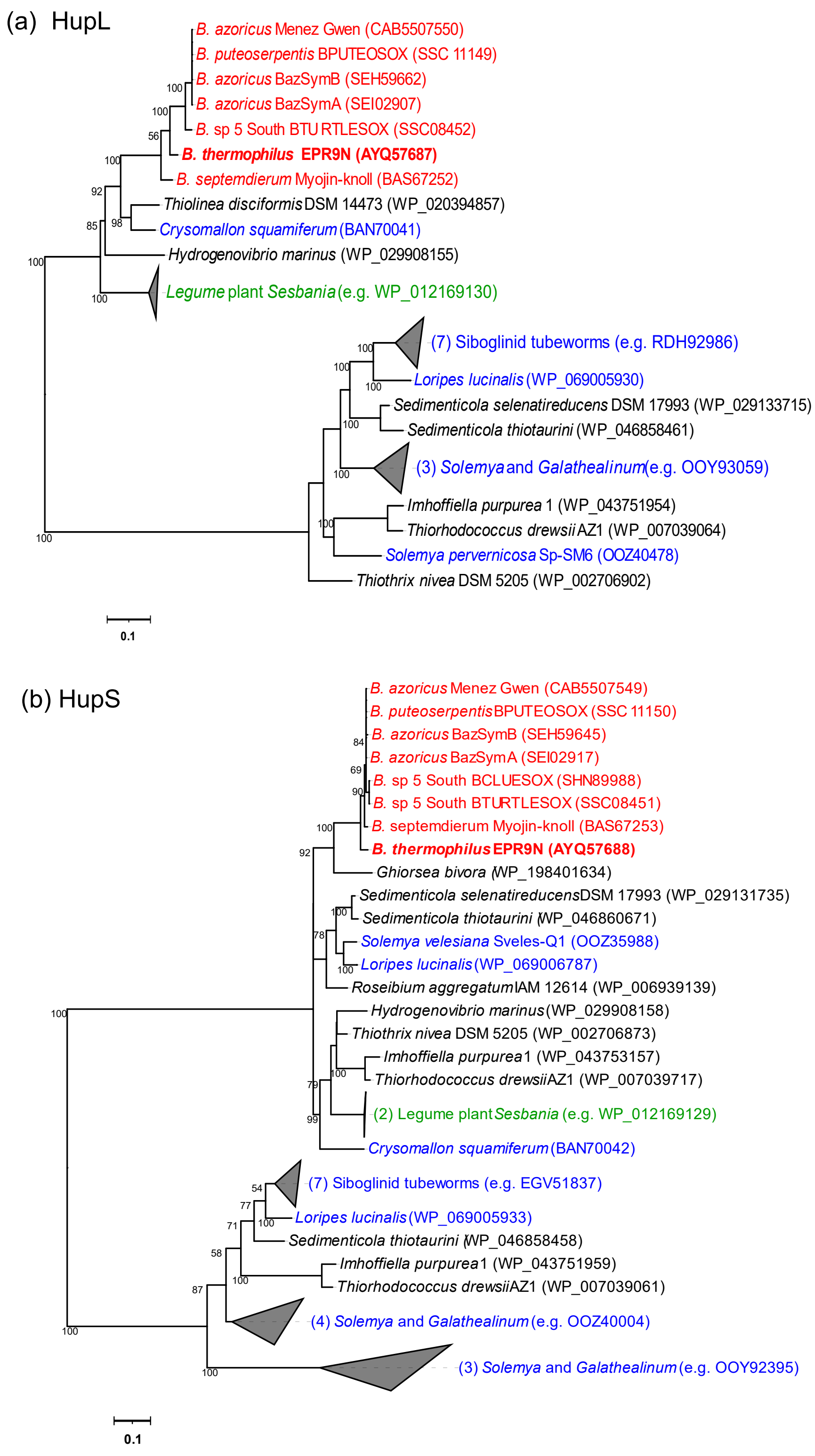
(a) HupL
B. azoricus Menez Gwen (CAB5507550)
B. puteoserpentis BPUTEOSOX (SSC 11149)
B. azoricus BazSymB (SEH59662)
B. azoricus BazSymA (SEI02907)
B. sp 5 South BTU RTLESOX (SSC08452)
B. thermophilus EPR9N (AYQ57687)
B. septemdierum Myojin-knoll (BAS67252)
Thiolinea disciformis DSM 14473 (WP_020394857)
Crysomallon squamiferum (BAN70041)
Hydrogenovibrio marinus (WP_029908155)
Legume plant Sesbania (e.g. WP_012169130)
(7) Siboglinid tubeworms (e.g. RDH92986)
Loripes lucinalis (WP_069005930)
Sedimenticola selenatireducens DSM 17993 (WP_029133715)
Sedimenticola thiotaurini (WP_046858461)
(3) Solemya and Galathealinum (e.g. OOY93059)
Imhoffiella purpurea 1 (WP_043751954)
Thiorhodococcus drewsii AZ1 (WP_007039064)
Solemya pervernicosa Sp-SM6 (OOZ40478)
Thiothrix nivea DSM 5205 (WP_002706902)
0.1
(b) HupS
B. azoricus Menez Gwen (CAB5507549)
B. puteoserpentis BPUTEOSOX (SSC 11150)
B. azoricus BazSymB (SEH59645)
B. azoricus BazSymA (SEI02917)
B. sp 5 South BCLUESOX (SHN89988)
B. sp 5 South BTURTLESOX (SSC08451)
B. septemdierum Myojin-knoll (BAS67253)
B. thermophilus EPR9N (AYQ57688)
Ghiorsea bivora (WP_198401634)
Sedimenticola selenatireducens DSM 17993 (WP_029131735)
Sedimenticola thiotaurini (WP_046860671)
Solemya velesiana Sveles-Q1 (OOZ35988)
Loripes lucinalis (WP_069006787)
Roseibium aggregatum IAM 12614 (WP_006939139)
Hydrogenovibrio marinus (WP_029908158)
Thiothrix nivea DSM 5205 (WP_002706873)
Imhoffiella purpurea 1 (WP_043753157)
Thiorhodococcus drewsii AZ1 (WP_007039717)
(2) Legume plant Sesbania (e.g. WP_012169129)
Crysomallon squamiferum (BAN70042)
(7) Siboglinid tubeworms (e.g. EGV51837)
Loripes lucinalis (WP_069005933)
Sedimenticola thiotaurini (WP_046858458)
Imhoffiella purpurea 1 (WP_043751959)
Thiorhodococcus drewsii AZ1 (WP_007039061)
(4) Solemya and Galathealinum (e.g. OOZ40004)
(3) Solemya and Galathealinum (e.g. OOY92395)
0.1

Figure S3. Phylogenies of hydrogenases (a) HupL and (b) HupS genes. Phylogenetic trees were inferred by using the maximum likelihood method with bootstrap values on the tree branches (only over 50 are shown). The taxa in green are of the symbionts of legume host *Sesbania*, in blue are the symbionts of marine invertebrates, and in bold red are the symbiont of *B. thermophilus* EPR9N. GenBank accession numbers of proteins are presented in parentheses.

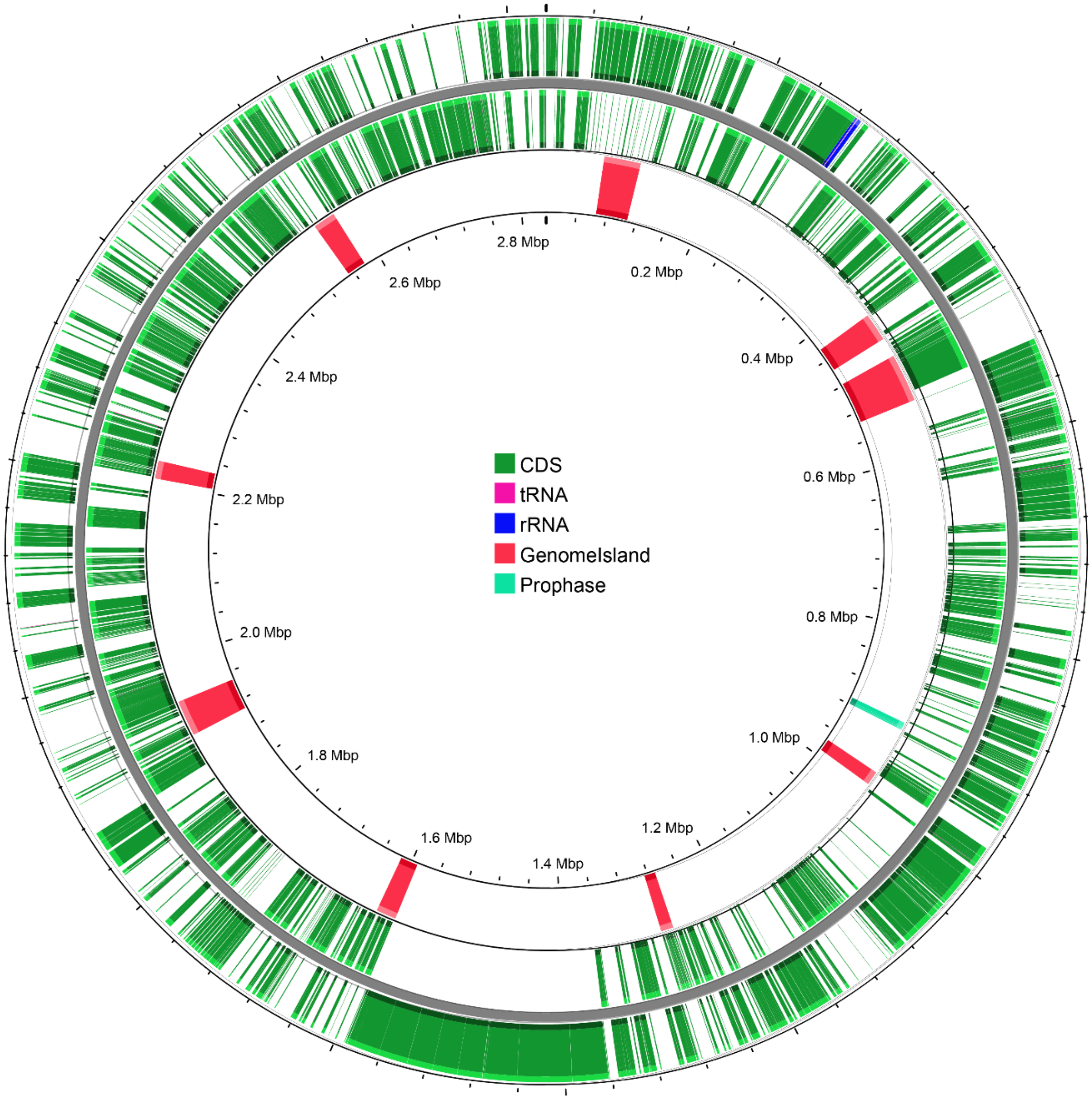

Figure S4. Genomic islands, and prophage regions in *B. thermophilus* EPR9N genome.
